# Phenotypic and Genotypic Characterization of Enterotoxigenic *Escherichia coli* Clinical Isolates from Northern Colombia, South America

**DOI:** 10.1155/2014/236260

**Published:** 2014-04-30

**Authors:** Julio A. Guerra, Yesenia C. Romero-Herazo, Octavio Arzuza, Oscar G. Gómez-Duarte

**Affiliations:** ^1^Division of Pediatric Infectious Diseases, Vanderbilt University School of Medicine, 1161 21st Avenue South, Nashville, TN 37232-2581, USA; ^2^Departamento de Microbiología, Grupo de Microbiología Clínica y Ambiental, Universidad de Cartagena, Cartagena, Colombia

## Abstract

Enterotoxigenic *Escherichia coli* (ETEC) are major causes of childhood diarrhea in low and middle income countries including Colombia, South America. To understand the diversity of ETEC strains in the region, clinical isolates obtained from northern Colombia children were evaluated for multiple locus sequencing typing, serotyping, classical and nonclassical virulence genes, and antibiotic susceptibility. Among 40 ETEC clinical isolates evaluated, 21 (52.5%) were positive for LT gene, 13 (32.5%) for ST gene, and 6 (15%) for both ST and LT. The most prevalent colonization surface antigens (CS) were CS21 and CFA/I identified in 21 (50%) and 13 (32.5%) isolates, respectively. The *eatA*, *irp2*, and *fyuA* were the most common nonclassical virulence genes present in more than 60% of the isolates. Ampicillin resistance (80% of the strains) was the most frequent phenotype among ETEC strains followed by trimethoprim-sulfamethoxazole resistance (52.5%). Based on multiple locus sequencing typing (MLST), we recognize that 6 clonal groups of ETEC clinical isolates circulate in Colombia. ETEC clinical isolates from children in northern Colombia are highly diverse, yet some isolates circulating in the community belong to well-defined clonal groups that share a unique set of virulence factors, serotypes, and MLST sequence types.

## 1. Introduction

Enterotoxigenic* Escherichia coli* (ETEC) are important enteric pathogens worldwide, especially affecting children in developing countries [1, 2]. ETEC strains are responsible for ~400 million diarrheal cases annually in children less than 5 years of age, resulting in 300,000 to 500,000 deaths, and they are the most common causes of traveller's diarrhea, accounting for 50% of all traveler's diarrhea episodes [3, 4]. ETEC strains belong to a highly diverse group of strains with respect to enterotoxin type, colonization surface antigens (CSs), serotypes, and ancestral lineages [5–8].

ETEC strains are defined by the presence of plasmid-encoded heat-labile toxin (LT) and/or the heat-stable toxins (ST) [9, 10]. ST, a guanylin homologue expressed in intestinal cells, is a heterogeneous peptide with two major subtypes STa, present predominantly in human ETEC isolates, and STb, present predominantly in animal ETECs [11]. Both subtypes induce diarrhea in piglets [12]. STa is further subdivided in two variants, STh and STp (from their initial detection among pigs) that have been reported in ETEC clinical isolates from different parts of the world [11, 13]. ETEC strains also express plasmid- or chromosomally encoded colonization surface antigens (CSs). These heterogeneous pili or nonpili surface structures are believed to promote small intestine ETEC colonization and they are currently considered important vaccine targets. Twenty-two different CSs have been identified among human ETEC of diverse geographic origins [14, 15]. ETEC isolates may produce one or more CSs, while some isolates do not express any or do not produce recognizable CSs [2]. LT and ST toxin types and CSs profiles from clinical ETEC isolates vary from one geographic region to another [2, 6, 16, 17].

ETEC also expresses a variety of nonclassical virulence factors that may be essential for pathogenicity and promising vaccine targets. Among nonfimbrial adhesins/invasins, Tia is a 25 kD outer membrane protein that interacts with host cell surface proteoglycans and by itself is sufficient to promote bacterial adherence and epithelial cell invasion when cloned into laboratory* E. coli* strains [18, 19]. The labile enterotoxin output gene (leoA), encoding a cytoplasmic protein with GTPase activity, is required for maximal LT secretion. Both Tia and LeoA are encoded in a 46-Kb pathogenicity island (Tia-PAI) [20, 21]. The TibA protein encodes a glycosylated autotransporter that mediates adhesion to surface epithelial cells, autoaggregation, and biofilm formation [22, 23]. The* etpBAC* locus encodes three proteins: EtpA, a 170 kDA secreted glycoprotein, EtpB a transport pore, and EtpC, a putative glycosyltransferase required both for optimal secretion and glycosylation of EtpA. The EtpA glycoprotein appears to act as a molecular bridge, binding the exposed regions of FliC at the flagellar tip and host surface structures [24, 25]. EatA, a serine protease autotransporter of the Enterobacteriaceae (SPATE) family, was shown to increase ETEC virulence in an animal model, by degrading mucin and facilitating LT release [25, 26]. Finally, the* irp*2 and* fyuA* genes, located in the high-pathogenicity island (HPI), encode a yersiniabactin-like iron scavenging system [27].

ETEC is the leading cause of diarrhea in children less than 5 years of age in Colombia, South America [28, 29], yet no information is available of the phenotypes and genotypes associated with these strains. The objectives of this study were to identify the most common genotypes associated with Colombian ETEC clinical isolates with respect to enterotoxins, CSs, nonclassical virulence genes, and genomic profiles and to determine the most common ETEC O:H serotypes and antimicrobial susceptibility patterns. Recognizing the most frequent circulating strains including the most common potential antigens may help prioritize ETEC diarrhea prevention measures including vaccine development research strategies.

We found that ETEC isolates were positive for LT, ST, or both ST/LT genes, the most prevalent CSs were CS21 and CFA/I, and the most common nonclassical virulence genes were* eatA*,* irp2,* and* fyuA*. Based on MLST, serotyping, and virulence genotype, Colombian ETEC clinical isolates showed broad genetic diversity, yet 6 distinctive clonal groups were identified.

## 2. Materials and Methods

### 2.1. Strains Used in This Study

Thirty-two ETEC clinical isolates from children less than 5 years of age with diarrhea and 8 ETECs obtained from healthy children from two studies were used. Seven of these ETEC strains came from children with diarrhea from a prevalence study previously described in two Caribbean cities in Colombia [29]. The remaining ETEC strains were obtained from case-control studies of children less than 5 years of age also in Cartagena, Colombia [28]. Twenty-five strains were from cases and 8 strains from healthy controls (Table S1). All ETEC clinical isolates identified from the two epidemiological studies mentioned above were included in the present study. These isolates were identified by multiplex PCR using ST primers (ST.F-5′GCTAAACCAGTA(G/A)GGTCTTCAAAA3′ and ST.R- 5′CCCGGTACA(G/A)GCAGGATTACAACA 3′) and LT primers (LT.F- 5′GCACACGGAGCTCCTCAGTC-3′ and LT.R- 5′TCCTTCATCCTTTCAATGGCTTT 3′) [29]. Reference ETEC and non-ETEC strains were used as positive and negative controls for all assays and they are described in Table 1. ETEC strains carrying different CS genes, used as controls for PCR detection assays, were kindly provided by Dr. Steven Savarino from the Naval Medical Research Center.

### 2.2. DNA Techniques

Unless otherwise specified, standard methods were used for plasmid isolation, genomic DNA isolation, and agarose electrophoresis DNA separation [30].* E. coli* clinical isolates were processed for isolation of genomic DNA as previously described [31]. In brief, overnight liquid cultures were centrifuged, and the pellet was resuspended in water, boiled for 10 min, and centrifuged again. The supernatant containing a crude DNA extract was used as a DNA template for PCR assays.

### 2.3. DNA Amplification

Detection of STh, STp, and LT toxin genes and 19 CSs genes was performed by multiplex polymerase chain reaction (mxPCR) assays as described before [32]. CSs genes tested included CFA/I, CS1, CS2, CS3, CS4, CS5, CS6, CS7, CS8, CS12, CS13, CS14, CS15, CS17, CS18, CS19, CS20, CS21, and CS22. Confirmatory single PCR was performed on strains positive on the multiplex PCR assays. Detection of* Tia*,* LeoA*,* TibA*,* EatA*,* EtpA*,* EtpB*,* FyuA*, and* Irp2 *genes was done by single PCR assays as described before [33].

### 2.4. Multilocus Sequence Typing (MLST)

Genetic diversity of ETEC strains was analysed by multilocus sequence typing (MLST) by using the University College Cork* E. coli* MLST scheme (http://mlst.warwick.ac.uk), which is based on sequencing of internal regions of 7 housekeeping genes* adk, fumC, gyrB, icd, mdh, purA,* and* recA* [34]. Phylogenetic trees were constructed using the Phylogeny.fr software available online at http://www.phylogeny.fr/version2_cgi/index.cgi by the cluster W method [35]. Forty seven-gene DNA sequence concatamers for each ETEC strain as well as 4* E. coli* control sequence concatamers from ancestral groups A, B1, B2, and D were assembled and aligned using the ClustalW program. The phylogenetic tree using the PhyML program was constructed using bootstrapping procedure (100 straps) [35, 36]. Some ETEC strains were assigned to clonal groups. A clonal group was defined as a group of more than one ETEC strains that do not seem to share ancestral origin with other ETEC and that have at least 2 strains with identical MLST DNA sequence.

### 2.5. Serotyping

Serotyping was performed at the* E. coli *Reference Center, Pennsylvania State University, according to standard methods for determining the O antigen [37]. H typing was performed using a fliC PCR-RFLP method [38].

### 2.6. Antimicrobial Susceptibility Testing

Antimicrobial susceptibility to 12 different antibiotics was tested using BD BBL Sensi-Disc Susceptibility Test Discs methods (Becton, Dickinson andCompany. © 2012 BD). Strain activity was tested against cefazolin (CZ), ceftriaxone (CRO), ampicillin (AM), amoxicillin/clavulanic acid (AMC), ceftazidime (CAZ), cefuroxime (CMX), cefepime (FEP), ciprofloxacin (CIP), gentamicin (GM), Meropenem (MEM), sulfamethoxazole (STX), and piperacillin tazobactam (TZP).

## 3. Results

### 3.1. Colombian ETEC Clinical Isolates Carry LT, ST, and LT/ST Enterotoxins

ETEC clinical isolates from children with diarrhea and with no diarrhea previously described were evaluated for the presence of LT and/or ST enterotoxin genes (See Table S1) [28, 29]. As shown in Table 2, LT-containing ETEC strains were the most frequently detected group (52.5%) followed by ST-containing ETEC strains (32.5%) and ETEC-LT/ST strains (15%). All ETEC-ST strains tested were positive for the STh variant. No STp variants were identified.

### 3.2. CSs Were Frequently Found among ETEC Clinical Isolates

CSs are piliated and nonpiliated structures believed to be involved in ETEC colonization of the human gut. In our study 75% of all clinical isolates were positive for at least one CS and 25% were negative for any CS (Table 2). Among ETEC-associated CS described in the literature, 10 different types of CSs were detected among Colombian ETEC isolates. Alone or in association, the most prevalent ETEC CSs were CS21, CFA/I, CS6, and CS5, present in 50.0%, 32.5%, 20%, and 12.5%, respectively.

Detection of CS21 was present in 12 (92.3%) out of 13 ETEC strains containing ST, followed by 7 (33.3%) out of 21 ETEC strains containing LT and only 1 (16.6%) out of 6 ETEC strains containing LT and ST toxins. CFA/I was found in 12 (92.3%) out of 13 ETEC strains containing ST strains and only in 1 (4.7%) out of 21 ETEC strains carrying LT. CFA/I was not detected among ETEC containing ST and LT toxins. CS6 was found in 4 (19%) out 21 LT-containing ETEC strains and in 4 (66.6%) out of 6 ST and LT containing ETEC strains. CS5 was only found among 5 (83.3%) out 6 ST/LT-containing ETEC strains. CS1, CS2, CS7, CS12, CS13, and CS17 CSs were only detected among LT-containing ETEC strains. Among CS-negative ETEC, 9 out 21 (42.8%) were LT-containing ETEC strains, and only 1 out of 12 (8.3%) were ST-containing ETEC strains.

CFA/I was detected in association with CS21 (Table 2) in 13 ETEC strains, 12 strains of them containing ST and one LT. No ETEC with ST/LT combination contained this CS pattern. The CS5-CS6 pattern was the second most common CSs combination present in 4 out of 40 ETEC strains (10.0%), all of them positive for ST and LT ETEC strains. The CS21-CS6 was the third most common CSs pattern only present in 3 out of 42 ETEC strains (7.1%), all positive for LT.

### 3.3. Nonclassical Virulence Genes Were Detected among Colombian ETEC Isolates

**Figure 1:**
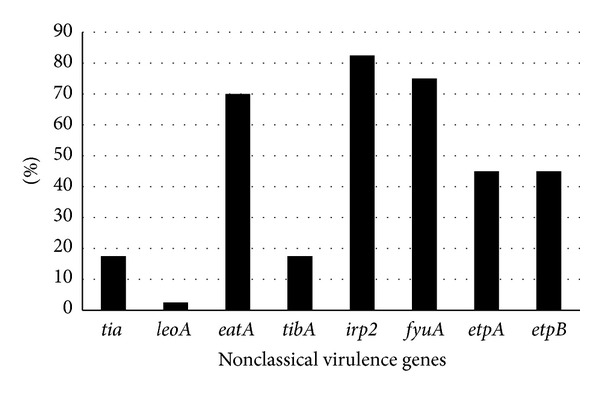
Proportion of nonclassical virulence factor genes among Colombian ETEC clinical isolates. Detection of nonclassical virulence factors was performed by nonvirulence genes PCR amplification of genomic DNA from Colombian ETEC clinical isolates as described in materials and methods.

Nonclassical virulence factors, believed to essential contributors to the pathogenesis of ETEC diarrhea, include factors associated with adherence, invasion, enterotoxin secretion, and iron acquisition. The* irp2*,* fyuA, *and* eatA* genes, present in 33 (82.5%), 30 (75%), and 29 (72.5%) ETEC isolates, respectively, were the most frequently detected nonclassical virulence ETEC genes (Figure 1). In contrast, strains carrying the* tia*-PAI-associated genes were uncommon. Only 1 (2.5%) strain was positive for the* leoA* gene and 7 (17.5%) for the* tia* gene. The* etpA* and* etpB* genes encoded by the same plasmid that harbors genes for toxins and CFA/I in strain H10407 were both detected in 18 (45%) ETEC isolates. The* tibA* gene was only detected in 7 (17.5%) ETEC isolates.

### 3.4. Phylogenetic and MLST Results

**Figure 2:**
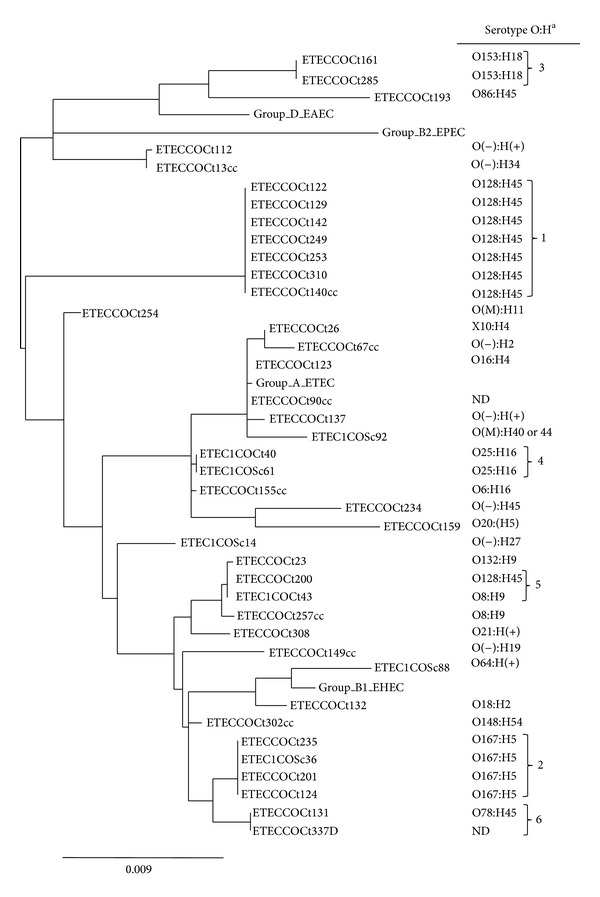
MLST phylogenetic tree and serotyping analyses of Colombian ETEC clinical isolates. Phylogenetic tree constructed after assembly and alignment of MLST DNA sequences using the ClustalW program. MLST and serotyping experiments are described in Materials and Method.* E. coli* strains from ancestral groups A, B1, B2, and D were used as control for phylogenetic analyses. EAEC:* enteroaggregative E. coli*; EPEC:* enteropathogenic E. coli*; ETEC:* enterotoxigenic E. coli*; STEC:* shiga toxin-producing E. coli*. (−) refers to negative reaction with standard antisera and/or PCR amplification. (+) refers to positive reaction; the group is novel and does not match known reference standards. (M) Multiple positives. (X) Unclassified O types. (ND) Not done. Numbers 1 to 6 correspond to clonal groups based on identical MLST DNA sequences.

To evaluate the genetic relatedness among Colombian ETEC isolates and with ancestral* E. coli* strains, MLST was conducted and a phylogenetic tree was analyzed. MLST sequences from* E. coli* pathogens representing ancestral* E. coli* groups A, B1, B2, and D were included in the analysis as controls. The phylogenetic tree constructed from Colombian ETEC MLST sequences is highly diverse as demonstrated by the extended branching (Figure 2). Despite genetic diversity, most ETEC strains seem to be derived from* E. coli *ancestral groups A and B1, as determined by the phylogenetic tree evolutionary relationships using* E. coli *control strains MLSTs from ancestral groups A, B1, B2, and D. Only a minority of the ETEC strain MLSTs were associated with* E. coli* strain MLSTs from phylogenic groups B2 and D.

Nineteen (47.5%) ETEC strains are clustered into six allelic groups. Based on identical MLST sequences within each cluster, we have designated these clusters as clonal groups 1 to 6. Based on evolutionary relationships using* E. coli* control strain MLSTs, clonal groups 1, 2, 4, 5, and 6 are associated with ancestral groups A and B1, while clonal group 3 MLST is associated with ancestral group D and B2. Analysis of MLST sequence types (SeqT) found that all 40 ETEC isolates have one designated specific SeqT (Table 3). SeqT is defined as the allelic profile resulting from the seven alleles assigned to each one of the 7 house-keeping loci sequences. In this study, we are reporting three new SeqTs, SeqT 4238, SeqT 4239, and SeqT 4252, for strains COCt26, COCt234, and COCt159, respectively. As part of the MLST new SeqT 4252, we have also reported a new* icd* gene DNA sequence designated* icd454* for the COCt159 ETEC strain. Twenty Colombian ETEC strains had MLST SeqT previously reported as ETEC. The most common MLST SeqT among Colombian ETECs isolates was the 2332 found in 7 (17.5%) ETEC isolates (Table 3). Five ETEC isolates had MLST SeqT previously observed among non-ETEC pathotypes while12 ETEC isolates had SeqT not previously observed among* E. coli* pathotypes.

### 3.5. Colombian ETEC Isolates Belong to a Highly Diverse Group of O:H Serogroups

O:H serogroups identification was conducted to determine the most common Colombian ETEC serotypes. Twenty-nine (72.5%) ETEC strains belonged to 16 different O serogroups (Figure 2). The most common O groups were O128 and O167 present in 8 (28.6%) and 4 (14.3%) of isolates, respectively. In addition, 7 ETEC isolates with rough colonies lacked side chains thus were classified as nontypeable with respect to O antigen. Thirty-four (85%) of ETEC isolates belonged to 13 different H types. The most common H serogroups were H45 type present in 11 (27.5%) ETEC isolates. Other H serogroups identified in 3 or more strains included H5 and H16. Five (14.7%) strains were H5 and 3 (8.8%) were H16. The most common O:H combination identified was the O128:H45 serotype present in 8 (20%) of the strains and 7 of them within the same MLST clonal group. Serogroup O167:H7 was present in 4 (10%) ETEC isolates that belong to clonal group 2. Less frequent serotypes combinations associated with MLST-based clonal groups included O153:H18 (clonal group 3) and O25:H16 (clonal group 4).

### 3.6. Colombian ETEC Isolates Have Low Level of Antibiotic Resistance

To evaluate ETEC clinical isolates for antibiotic susceptibility conventional disk antibiograms were performed (Table 4). Ampicillin, trimethoprim-sulfamethoxazole, cefazolin, and amoxicillin clavulanate resistance was detected among 27 (67.5%), 20 (50%), 6 (15%), and 2 (5%) isolates, respectively. No resistance to ceftriaxone, ceftazidime, cefepime, ciprofloxacin, and piperacillin/tazobactam was detected among ETEC isolates. Resistance to gentamicin was detected in one strain only.

### 3.7. Features Shared among ETEC Clonal Groups Including O:H Serogroup, Classical and Nonclassical Virulence Genes, and Antibiotic Resistance Profile

Nineteen (47.5%) Colombian ETEC isolates belong to 6 clonal groups. Clonal group 1 contained 7 ETEC isolates defined by identical MLST sequence and sequence type (2332). All clonal group 1 ETECs had identical O:H serotype and virulence genes (STh enterotoxin, CS21-CFA/I CSs and non-classical virulence factors) and they were resistant to ampicillin and sulfamethoxazole (Table 5). Strains in clonal group 1 were isolated from different individuals at different year periods. ETEC isolates from clonal group 2 with 4 share the same serotype as well as classical and nonclassical virulence factors. Three of them were resistant to ampicillin and sulfamethoxazole. Clonal groups 3 and 4 contain only two ETEC strains each with the same serotype. Clonal groups 5 and 6 do not seem to share the same serotype.

## 4. Discussion

ETEC diarrhea is a leading cause of morbidity and mortality in children less than 5 year of age living in underserved geographic areas of the world and a leading cause of traveler's diarrhea. ETEC is also a leading cause of morbidity in Colombia, a middle income country in Latin America. In this study, we show that northern Colombian ETEC clinical isolates from children less than 5 years of age are a highly diverse group of strains based on MLST, serotyping, and presence of classical and nonclassical virulence factors, yet 6 clonal groups were identified. A limitation of our study is the limited number of ETEC strains tested and also the strains origin is limited to two mayor urban centers in northern Colombia. Accordingly we will confine our analysis and conclusions to northern Colombian ETEC strains. Further studies will be necessary to evaluate ETEC diversity from all Colombian corners to better define ETEC Colombian virulence and colonization gene diversity and strain clonality.

LT, the most frequently toxin type found in the Colombian ETEC isolates, was detected in 67.5% of strains, whether alone or in combination with ST [41]. This entorotoxin pattern was also reported in Bangladesh and Peru, where LT-producing ETEC was seen in 52% and 72% of the cases, respectively [42, 43]. This is in contrast with other studies from Indonesia and Chile showing that ST-producing ETEC predominated [33, 44]. ST enterotoxin variants STp and STh induce disease in humans, and differentiation of STp from STh may help identify differences in the epidemiology of these two strains [45]. In this study, all Colombian ETEC strains positive for ST were only positive for the STh variant; no ETEC strains positive for STp were identified. Low frequency of STp ETEC strains was also reported among Brazilian, Bolivian, and Chilean ETEC clinical isolates suggesting that STh toxin predominates among Latin American ETECs [33, 46, 47].

CS21 was the most commonly identified CS among Colombian ETEC isolates, followed by CFA/I and CS6. The association of CS21 and CFA/I among ETECs was also remarkable. Similar findings were reported in Chile where CS21 was found in association with CFA/I [33]. In contrast, CFA/I and CS14 predominated among Bolivian ETEC strains [46]. CS21 has also been identified in ETEC strains from Argentina, Brazil, Bolivia, Egypt, and Bangladesh [33, 46, 48–51].

CS21 is long rod-like fimbria that directs adhesion to intestinal epithelial cells and mediate self-aggregation and twitching motility, and it is involved in pathogenesis [52–54]. In this study, CS21 was more often associated with LT producer Colombian ETEC strains. ETEC strains expressing CS21 tend to be isolated in higher proportion among pediatric populations [50]. It is likely that the higher proportion of CS21 among Colombian ETECs is due to the fact that all ETEC strains were obtained from children less than 5 years of age. CFA/I, CS6, and CS21 were detected among all ETEC toxins profiles, demonstrating their extended distribution. Similarly, CFA/1, CS6, and CS21 have been widely distributed among ETEC strains worldwide [2, 14]. In 10 (25%) of Colombian ETEC isolates, no CSs were identified. This indicates that these 10 ETECs do not express any known CSs, they contain CSs variants unable to be recognized by conventional PCR, or they contain unknown CSs unable to be recognized with current PCR assays [2, 55]. These strains may express novel CSs pending to be identified.

Nonclassical virulence genes were detected among Colombian ETEC strains. The* eatA*,* irp2,* and* fyuA* were the most frequent. These genes are known to be associated with the ETEC HPI pathogenicity island [27]. The distribution of nonclassical virulence genes among Colombian ETEC strains is similar to Chilean ETEC strains, except that etpA and etpB are present at lower rates (18%) [33]. The* tia *and* leoA *genes were also detected at low frequency, similar to the reported frequency among strains from Bolivia, Chile, Guatemala, Mexico, and India [33, 41, 56]. In contrast,* irp2 *and* fyuA *genes were detected in more than 70% of the strains as previously described among Chilean strains [33].

Colombian ETEC strains have a widely diverse phylogenetic distribution represented in 17 known MLST sequence types. Ample diversity among human ETEC strains from different geographic regions worldwide was reported previously [8]. Colombian ETECs segregated with any ancestral* E. coli* clonal groups A, B1, B2, and D. This is consistent with the idea that ETEC strains are representative members of distinct ETEC lineages [8, 10]. In regard to serotyping, 16 different O groups and 13 H groups were detected among Colombian ETECs. Studies on ETEC strains from different regions of the world have reported 78 different O serogroups and 34 H serogroups, indicating that ETEC serotype diversity among Colombia ETEC is similar to serotype diversity reported in elsewhere [57].

Despite serogroup diversity, there is an association between serogroups and genetic linkages as demonstrated by the serotype-specific clonal clusters among ETEC Brazilian strains [58]. In our study, 6 clonal groups were identified based on MLST and some of them shared similar serotypes, CSs, and nonclassical virulence genes. The MLST sequence types for each of clonal groups 1 to 6 that had been described before in the* E. coli* MLST database suggest that these clonal groups may circulate not only in Colombia but also in other geographic regions. The Colombian ETEC clonal group 1 with MLST SeqT2332 was associated with O128 serogroup. This serotype was previously described among ETEC strains in Bangladesh, Brazil, Egypt, and Tunisia [7, 58–60]. The SeqT2332 was described in Mexico according to the University College Cork* E. coli *MLST Database, yet it is not frequently detected among Bolivian or Mexican ETEC strains. SeqT443 is the second most common sequence type among Colombian ETECs. SeqT423 and SeqT443 predominate in Mexican ETEC isolates while SeqT398 predominates in Guatemala and Mexico ETEC strains [61]. While limited studies are available in Latin America on phylogeny of ETEC clinical isolates using MLST schemes, it is suggested based on the available data that ETEC are highly diverse in Latin America. This study has tested a limited number of ETEC strains and the origin of these isolates is also limited to two mayor urban centers in northern Colombia. Accordingly, we confine our analysis and conclusions to Northern Colombian ETEC strains. Further studies are necessary to evaluate ETEC diversity from all Colombian corners to better define Colombian ETEC genotype, phenotype, clonality, and genetic diversity.

## 5. Conclusion

In summary, ETEC clinical isolates from northern Colombia are a highly diverse group of intestinal pathogens that possess multiple combinations of classical and nonclassical virulence factors as well as MLST sequence types and serotypes. Despite the genotypic and phenotypic diversity, 6 well-defined clonal groups were identified. These predominant clonal groups have been circulating within the community for several years and they share an almost identical set of classical and nonclassical virulence factors as well as MLST and serotype. Further research in Colombia and other Latin American countries is necessary to identify the most prevalent ETEC-associated virulence factors with immunoprotection potential. Promising ETEC vaccine candidates should take into account predominant antigens, antigenic diversity, and geographic variation.

## Supplementary Material

The supplementary table S1 provides the complete list of ETEC clinical isolates used in the present study. This table includes information on source, date and place of strain isolation, as well as data on virulence genes (enterotoxins, CSs and non-classical virulence factors), serotype, MLST sequence type and antimicrobial susceptibility.

## Figures and Tables

**Table 1:** ETEC and non-ETEC reference strains used as controls in PCR assays.

Type	Strain	Serotype	Toxin type	CS type	Source
ETEC	E24377A	O139:H28	*LT/ST *	*CS1, CS3 *	Levine et al., 1984 [39]
ETEC	910980-2	*O25:NM *	*STh *	*CS4, CS6 *	NMRC^b^
ETEC	W6520A	*O114:H49 *	*LT *	*CS7 *	NMRC
ETEC	WS6866B-2	*NK *	*LT *	*CS8 (i.e., CFA/III) *	NMRC
ETEC	M421C1	*NK *	*LT/STh *	*CS5, CS6 *	VU^c^
ETEC	WS6474D	*O68:H12 *	*LTST *	*CS12 (i.e., PCFO159) *	NMRC
ETEC	911205	*O64:NM *	*LT *	*CS13 (i.e., PCFO9) *	NMRC
ETEC	E7476A	*O166:H27 *	*STh *	*CS14 *	NMRC
ETEC	8786	*O117:H4 *	*ST *	*CS15 (i.e., 8786) *	NMRC
ETEC	WS6788A	*O8:H9 *	*LT *	*CS17 *	NMRC
ETEC	ARG-2	*O20:H− *	*LT *	*CS18 (i.e., PCFO20) *	NMRC
ETEC	DS26-1	O8:H9	LT	CS19	NMRC
ETEC	WS7179A-2	O17:H45	LTST	CS20	NMRC
ETEC	H10407^a^	O78:H11	LT/STh-STp	CFA/I	Evans et al., 1975 [40]
ETEC	145C2	NK	LT/STh	CS2, CS3, CS21	VU
ETEC	E9034A	NK	LT/STh	CS3/CS21	Levine et al., 1984 [39]
*E. coli *	DH5*α*		None	None	VU

^a^Strain used as a positive control for nonclassical virulence factors.

^b^NMRC refers to Naval Medical Research Center.

^c^VU refers to Gomez-Duarte's laboratory collection at Vanderbilt University.

NK refers to not known.

**Table 2:** Distribution of LT and ST enterotoxins and colonization surface antigens among Colombian ETEC clinical isolates.

Toxin gene type	Total number of isolates^a^	CF type (s) produced	Number (%) of isolates
LT	21	CS21 + CFA/I	1 (4.7)
		CS21 + CS6	3 (14.2)
		CS21 + CS12	1 (4.7)
		CS21 + CS7	1 (4.7)
		CS21 + CS2 + CS3	1 (4.7)
		CS1	1 (4.7)
		CS7	1 (4.7)
		CS19	2 (9.5)
		CS6	1 (4.7)
		CF undetected^b^	9 (42.8)

LT-ST	6	CS21	1 (16.6)
		CS5 + CS6	4 (66.6)
		CS5	1 (16.6)

ST	13	CS21 + CFA/I	12 (92.3)
		CF undetected^b^	1 (8.3)

^a^ETEC isolates positive for any enterotoxin and positive or negative for any CSs.

^b^CSs undetected by PCR reaction.

**Table 3:** Distribution of MLST sequence types among Colombian ETEC isolates.

Origin of ETEC isolates number (%)			SeqT	Associated pathotype^a^
Child with diarrhea	Healthy child	Total		
6	1	7 (17.5)	SeqT2332	ETEC
1	0	1 (2.5)	SeqT849	ETEC
2	0	2 (5.0)	SeqT88	ETEC
1	0	1 (2.5)	SeqT100	ETEC
0	1	1 (2.5)	SeqT4	ETEC
0	1	1 (2.5)	SeqT94	ETEC
2	0	2 (5.0)	SeqT1312	ETEC
1	0	1 (2.5)	SeqT731	ETEC
1	1	2 (5.0)	SeqT10	ETEC, EAEC, EPEC, ExPEC
1	0	1 (2.5)	SeqT34	EAEC
2	0	2 (5.0)	SeqT38	EAEC
1	0	1 (2.5)	SeqT501	EAEC
0	1	1 (2.5)	SeqT23	EHEC
1	0	1 (2.5)	SeqT2066	Commensal
1	0	1 (2.5)	SeqT216	Commensal
1	0	1 (2.5)	SeqT3855	ETEC
2	0	2 (5.0)	SeqT641	Unknown
2	0	2 (5.0)	SeqT173	Unknown
1^b^	0	1 (2.5)	SeqT155	ETEC, EAEC, ExPEC
4^c^	0	4 (10.0)	SeqT443	Unknown
0	1^d^	1 (2.5)	SeqT1623	Unknown
1^e^	0	1 (2.5)	SeqT2067	Unknown
1	0	1 (2.5)	SeqT4238^f^	New SeqT
1	0	1 (2.5)	SeqT4239^f^	New SeqT
1	0	1 (2.5)	SeqT4252^g^	New SeqT

34 (85.0)	6 (15.0)	40 (100.0)		

^a^Associated pathotype according to MLST Databases at the ERI, University College Cork. ^b^Single mutation in *fumC* at position 158. ^c^Single mutation in *icd* at position 158. ^d^Single mutation in *purA* at position 260. ^e^Mutations in *icd* at position 110 and *fumC* at position 153. ^f^New sequence types submitted to the MLST Databases at the ERI, University College Cork. ^g^New SeqT submitted to MLST database; in addition, the new *icd *sequence for this strain was assigned number *icd* 454.

**Table 4:** Level of antibiotic resistance among Colombian ETEC clinical isolates.

Antibiotics	ETEC isolates number (%)		
	Susceptible	Intermediate	Resistant
Cefazolin (CZ)	29 (72.5)	5 (12.5)	6 (15.0)
Ceftriaxone (CRO)	40 (100)	0 (0)	0 (0)
Ampicillin (AM)	8 (20.0)	5 (15.0)	27 (67.5)
Amoxicillin/clavulanic acid (AMC)	25 (62.5)	13 (32.5)	2 (5.0)
Ceftazidime (CAZ)	40 (100)	0 (0)	0 (0)
Cefuroxime (CXM)	39 (97.5)	1 (2.5)	0 (0)
Cefepime (FEP)	40 (100)	0 (0)	0 (0)
Ciprofloxacin (CIP)	40 (100)	0 (0)	0 (0)
Gentamicin (GM)	39 (97.5)	0 (0)	1 (2.5)
Meropenem (MEM)	39 (97.5)	0 (0)	1 (2.5)
Sulfamethoxazole (STX)	17 (42.5)	1 (2.5)	22 (55.0)
Piperacillin/tazobactam (TZP)	40 (100)	0 (0)	0 (0)

Four ETECs resistant to a single antibiotic (AM); 15 resistant to 2 antibiotics (AM and STX), 7 ETEC resistant to 3 antibiotics (AM-STX-CZ or AM-STX-GM or AM-STX-AMC), and a single ETEC resistant to 4 antibiotics (AM, STC, CZ, and AMC).

**Table 5:** Characterization of the 6 ETEC clonal groups based on MLST, serotype, classical and nonclassical virulence factors, and antibiotic pattern.

Isolate	Classical virulence factors^a^			Non-classical virulence factors	Clonal group^e^	SeqT group^f^	Serotype	Antibiotic resistance^h^
	Enterotoxins		CS^c^				
	ST^b^	LT					
COCt122	+	−	CFA/I, CS21	*eatA*, *irp2*, *fyuA*, *etpA*, *etpB *	1	2332	O128:H45	AM, STX
COCt129	+	−	CFA/I, CS21	*eatA*, *irp2*, *fyuA*, *etpA*, *etpB *	1	2332	O128:H45	AM, STX
COCt142	+	−	CFA/I, CS21	*eatA*, *irp2*, *fyuA*, *etpA*, *etpB *	1	2332	O128:H45	AM, STX
COCt249	+	−	CFA/I, CS21	*eatA*, *irp2*, *fyuA*, *etpA*, *etpB *	1	2332	O128:H45	AM, STX
COCt253	+	−	CFA/I, CS21	*eatA*, *irp2*, *fyuA*, *etpA*, *etpB *	1	2332	O128:H45	AM, STX
COCt310	+	−	CFA/I, CS21	*eatA*, *irp2*, *fyuA*, *etpA*, *etpB *	1	2332	O128:H45	AM, STX, CZ
COCt140cc	+	−	CFA/I, CS21	*eatA*, *irp2*, *fyuA*, *etpA*, *etpB *	1	2332	O128:H45	AM, STX, AMC

COCt124	+	+	CS5, CS6	*eatA*, *tia *	2	443^g^	O167:H5	ND
COCt201	+	+	CS5, CS6	*eatA*, *tia *	2	443^g^	O167:H5	AM, STX
COCt235	+	+	CS5, CS6	*eatA*, *tia*, *irp2*, *fyuA *	2	443^g^	O167:H5	AM, STX
1COSc36	+	+	CS5, CS6	*eatA*, *tia *	2	443^g^	O167:H5	AM, STX

COCt 161	+	−	ND^d^	*irp2*, *fyuA *	3	38	O153:H18	AM, STX, CZ
COCt 285	+	−	CFA/I, CS21	*eatA*, *irp2*, *fyuA*, *etpA*, *etpB *	3	38	O153:H18	AM, STX

1COCt40	−	+	CS6, CS21	*eatA*, *irp2*, *fyuA *	4	1312	O25:H16	AM, STX, CZ
1COSc61	−	+	CS6, CS21	*eatA*, *irp2*, *fyuA *	4	1312	O25:H16	AM, STX, CZ

*COCt200 *	*+ *	*− *	*CS21*, *CFA/I *	*eatA*, i*rp2*, *fyuA*, *etpA*, *etpBirp2*, *fyuA *	*5 *	*88 *	*O128:H45 *	*AM, STX *
*1COCt43 *	*− *	*+ *	*CS18 *		*5 *	*88 *	*O8:H9 *	*AM, STX, CZ *

*COCt131 *	*− *	*+ *	*CS7*, *CS21 *	*eatA*, *tibA*, *irp2*, *fyuA*, *etpA*, *etpB *	*6 *	*137 *	*O78:H10 *	*AM, STX *
*COCt337 *	*− *	*+ *	*CS7 *	*eatA*, *tibA*, *irp2*, *fyuA*, *etpA*, *etpB *	*6 *	*137 *	*O(−):H(−) *	*AM, STX, CZ *

^a^ST, heat-stable toxin; heat-labile toxin.

^b^All ETEC TS positives were positive for the STh variant.

^c^CS refers to colonization surface antigens.

^d^ND: not detected.

^e^Based on phylogenetics (see Section 2).

^f^As determined by EcMLST (http://mlst.ucc.ie/mlst/dbs/Ecoli).

^g^ETEC strains with SeqT 443 that contain a single *icd* locus variant. The *icd* gene has 517/518 matches (mutation in T-229).

^h^Resistance to AM, ampicillin; STX: sulfamethoxazole; CZ: cefazolin; AMC: amoxicillin /clavulanic acid.
