# Supplementary material for: Phenotypic and Genotypic Characterization of Enterotoxigenic Escherichia coli Clinical Isolates from Northern Colombia, South America
Source: Biomed Res Int. 2014 Apr 30;2014:236260. doi: 10.1155/2014/236260 (PMC4022111; doi:10.1155/2014/236260)
Supplement: Supplementary file 1 — The supplementary table S1 provides the complete list of ETEC clinical isolates used in the present study. This table includes information on source, date and place of strain isolation, as well as data on virulence genes (enterotoxins, CSs and non-classical virulence factors), serotype, MLST sequence type and antimicrobial susceptibility. [file 236260.f1.pdf]

Table S1. Characterization of 40 ETEC clinical isolates based on clinical, genotypic, and phenotypic features.

ETEC strain	Age[a]	Date of Isolation	City of Isolation	Diarrhea	Toxin[b]	CS	Non Classical virulence genes	Clonal Group	SeqT[c]	O:H type	Antibiotic resistance[d]
COCt 023	4 y	10/5/2009	Cartagena	Yes	LT	ND	*tia/ irp2*		2066	132:28	AM, STX
COCt 026	2 y	11/5/2009	Cartagena	Yes	LT/ST	CS21	*eata/ tia/ etpA/ etpB*		4238[e]	X10:4	
COCt 112	2 m	29/52009	Cartagena	Yes	LT	ND	*ND*		641	(-):(+)	AM
COCt 122	2 y	30/5/2009	Cartagena	Yes	ST	CS21, CFA/I	*eata/ irp2/ fyuA/ etpA/ etpB*	1	2332	128:45	AM, STX
COCt 123	1 y	30/5/2009	Cartagena	Yes	LT	ND	*irp2/ fyuA*		10	16:4	AM, GM, STX
COCt 124	2 m	30/5/2009	Cartagena	Yes	LT/ST	CS5, CS6	*eata/ tia*	2	443[f]	167:5	
COCt 129	2 m	31/5/2009	Cartagena	Yes	ST	CS21/ CFAI	*eata/ irp2/ fyuA/ etpA/ etpB*	1	2332	128:45	AM, STX
COCt 131	5 m	31/5/2009	Cartagena	Yes	LT	CS7, CS21	*eatA/ tibA/ irp2/ fyuA/ etpA/ etpB*	6	173	78:10	AM, STX
COCt 132	7 m	31/5/2009	Cartagena	Yes	LT	ND	*fyuA*		847	X18:2	AM, STX
COCt 137	4 y	2/6/2009	Cartagena	Yes	LT	CS21/ CFAI	*eata/ irp2/ fyuA/ etpA/ etpB*		34	(-):(+)	
COCt 142	3 m	3/6/2009	Cartagena	Yes	LT	CS21/ CFAI	*eata/ irp2/ fyuA/ etpA/ etpB*	1	2332	128:45	AM,STX
COCt 159	2 y	11/6/2009	Cartagena	Yes	ST	CS21/ CFAI	*eatA/ irp2/ fyuA/ etpA/ etpB*		4252[e]	Neg(-):45	
COCt 161	2 y	11/6/2009	Cartagena	Yes	ST	ND	*irp2/ fyuA*	3	38	153:18	AM, CZ, STX
COCt 193	1 y	19/6/2009	Cartagena	Yes	LT/ST	CS21/ CFAI	*eata/ irp2/ fyuA/ etpA/ etpB*		501	86:45	AM
COCt 200	4 y	20/6/2009	Cartagena	Yes	LT/ST	CS21/ CFAI	*eata/ irp2/ fyuA/ etpA/ etpB*	5	88	128:45	
COCt 201	3 y	20/6/2009	Cartagena	Yes	LT/ST	CS5, CS6	*eatA/ tia*	2	443[f]	167:5	AM, STX
COCt 234	2 y	1/7/2009	Cartagena	Yes	LT/ST	CS5	*leoA/ eatA/ tia/ irp2/ fyuA*		3855	20:5	AM, AMC, CZ, STX
COCt 235	11 m	2/7/2009	Cartagena	Yes	LT/ST	CS5, CS6	*eatA/ tia/ irp2/ fyuA*	2	443[f]	167:5	AM, STX
COCt 249	4 m	9/7/2009	Cartagena	Yes	ST	CS21/ CFAI	*eata/ irp2/ fyuA/ etpA/ etpB*	1	2332	128:45	AM, STX
COCt 253	3 y	11/7/2009	Cartagena	Yes	ST	CS21/ CFAI	*eata/ irp2/ fyuA/ etpA/ etpB*	1	2332	128:45	AM, STX
COCt 254	4 y	11/7/2009	Cartagena	Yes	ST	CS21/ CFAI	*eata/ irp2/ fyuA/ etpA/ etpB*		4239[e]	M:11	AM
COCt 285	7 m	19/7/2009	Cartagena	Yes	ST	CS21/ CFAI	*eata/ irp2/ fyuA/ etpA/ etpB*	3	38	153:18	AM, STX
COCt 308	11 m	26/7/2010	Cartagena	Yes	LT	ND	*irp2*		155	21:(-)	
COCt 310	1 y	27/7/2009	Cartagena	Yes	ST	CS21/ CFAI	*eatA/ irp2/ etpA/ etpB*	1	2332	128:45	AM, CZ, STX
COCt 337	7 m	4/8/2009	Cartagena	Yes	LT	CS7	*eatA/ tibA/ irp2/ fyuA/ etpA/ etpB*	6	173	ND	AM, CZ, STX
COCt 013cc	4 y	21/7/2009	Cartagena	No	LT	CS12, CS21	*irp2/ fyuA*		641	(-):34	AM
COCt 067cc	4 y	16/12/2009	Cartagena	No	LT	ND	*irp2/ fyuA/ etpA/ etpB*		100	(-):2	
COCt 090cc	2 y	08/3/2010	Cartagena	No	LT	ND	*irp2/ fyuA*		10	ND	AM, STX

COCt 140cc	1 y	12/3/2010	Cartagena	No	LT	CS21/ CFAI	*eatA/ irp2/ fyuA/ etpA/ etpB*	1	2332	128:45	AM, AMC, STX
COCt 149cc	1 y	12/3/2010	Cartagena	No	LT	CS19	*irp2/ fyuA*		1623	(-):19	
COCt 155cc	3 y	12/3/2010	Cartagena	No	LT	CS3, CS21, CS2	*eata/ irp2/ fyuA/ etpA/ etpB*		4	6:16	AM, STX
COCt 275cc	2 y	17/4/2010	Cartagena	No	LT	CS1	*irp2/ fyuA*		23	8:9	
COCt 302cc	1 y	17/4/2010	Cartagena	No	LT	CS21, CS6	*irp2*		94	148:28	
1COCt 040	3 y	II[g]/2007	Cartagena	Yes	LT	CS21, CS6	*eatA/ irp2/ fyuA*	4	1312	25:16	AM, CZ, STX
1COCt 043	1 y	II/2007	Cartagena	Yes	LT	CS19	*irp2/ fyuA*	5	88	8:9	
1COSc 014	1 y	I[g]/2007	Sincelejo	Yes	LT	ND	*irp2/ fyuA*		731	(-):27	AM
1COSc 036	1 y	1/2007	Sincelejo	Yes	LT/ST	CS5, CS6	*eatA/ tiA*	2	443[g]	167:5	AM, STX
1COSc 061	1 y	I/2007	Sincelejo	Yes	LT	CS21, CS6	*eatA/ irp2/ fyuA*	4	1312	25:16	AM, CZ, STX
1COSc 088	1 y	I/2007	Sincelejo	Yes	LT	CS6	*irp2/ fyuA*		2067	64:(+)	STXi
1COSc 092	3 m	I/2007	Sincelejo	Yes	LT	ND	*ND*		216	M:40/44	

[a] Age: Child age at the time of specimen collection is given in years (y) or months (m).
[b] All ETEC tested positive for the STh variant.
[c] SeqT determined by EcMLST (http://mlst.ucc.ie/mlst/dbs/Ecoli).
[d] Resistance to AM, ampicillin; AMC, Amoxillin/clavulanic acid; CZ, cefazolin; GM: gentamicin; STX, sulfamethoxalone.
[e] New MLST SeqT.
[f] ETEC MLST sequence with a single locus variant in the *icd* gene with 517/518 matches (mutation in T-229). The variant otherwise corresponds to SeqT443.
[g] I or II refers to first or second semester.
ND indicate not detected.
